# The Histology-Driven Differential Diagnosis in Bowel Inflammatory Conditions Is Not All That Obvious: Evidence from a Survey Based on Digital Slides

**DOI:** 10.3390/diagnostics13243684

**Published:** 2023-12-18

**Authors:** Gabriella Canavese, Enrico Costantino Falco, Nuria Perez-Diaz-del-Campo, Gian Paolo Caviglia, Fabrizia Di Giovanni, Davide Giuseppe Ribaldone

**Affiliations:** 1 Department of Pathology, Città della Salute e della Scienza di Torino, 10126 Turin, Italy; enricoc.falco@gmail.com (E.C.F.);; 2 Department of Medical Sciences, University of Turin, 10126 Turin, Italygianpaolo.caviglia@unito.it (G.P.C.); davrib_1998@yahoo.com (D.G.R.)

**Keywords:** inflammatory bowel disease, histology, diagnosis, pattern, agreement

## Abstract

(1) Background: when the pathologist faces histologic slides from colonoscopies in daily practice, given the large number of entities and etiologies under inflammatory bowel conditions, in-depth definition of the histological spectrum and the recommendations of current guidelines are often not enough to conclusively define a diagnostic framework. Histological patterns should be organized hierarchically in flowcharts that consider the correlation with clinical data. We conducted an online survey asking a group of gastroenteropathologists to apply a pattern classification based on the most significant lesions in colitis differential diagnosis: crypt distortion and activity. (2) Methods: digital slides from 20 endoscopy samples were analyzed by twenty pathologists and classified according to the occurrence of crypt distortion (nondestructive–destructive colitis) and subsequently to the evidence of activity (ND1-2-3, D1-2). (3) Results: in 8 out of 20 (40%) cases, the participants reached a full agreement regarding the evaluation of crypt distortion (5 cases: nondestructive colitis; 3 cases: destructive colitis). The calculated agreement was k = 0.432. In the second-level quiz (ND1-2-3 and D1-2), full agreement between participants was achieved for 7 of the 28 (25%) possible classifications, with k = 0.229. (4) Conclusions: The findings from this survey are indicative of an unexpectedly low consensus, even among dedicated pathologists, about the recognition of histological changes that are commonly considered critical lesions in the histologic identification of bowel non-neoplastic diseases. In our opinion, these divergences imply a significant risk of misdiagnosis of bowel inflammatory conditions, hampering the usefulness of histological assessment.

## 1. Introduction

Bowel mucosa has a uniform reaction modality to pathogenic noxae and generates similar histologic changes despite different etiologies. This is a well-known troubling topic for pathologists committed to gastroenteric disease since histologic features of non-neoplastic disease are often barely specific and unfit for a precise diagnostic setting, requiring strong integration with clinical data, endoscopic evidence, and laboratory reports.

Morphologic spectrum and histologic criteria of differential diagnosis in the field of bowel non-neoplastic conditions have been described by various authors to improve the diagnostic classification of the inflammatory processes of bowel mucosa. Crypt distortion, mucosal disruption, basal plasmacytosis, and activity are currently adopted as the characteristic sign of inflammatory bowel disease (IBD), while the number of eosinophils, intraepithelial lymphocytes, and apoptotic bodies are among the most important parameters in the differential diagnosis between non-IBD chronic colitis [1,2,3].

Consensus papers have been drafted accordingly, and to avoid inadequacy of the necessary prerequisites for histologic examination, current guidelines provide instructions about the proper collection of clinical data, adequacy of sampling protocols, and biopsy specimens handling procedures, both in IBDs [4,5,6] and in non-IBD colitis [7].

Notwithstanding this, the interpretation of colitis histology is often tricky, given the large number of entities and etiologies under non-neoplastic bowel conditions and the inaccurate definition of the morphological features [8]; a study from our group demonstrated that guidelines recommendations are infrequently met, and the scarcely available studies on diagnostic reproducibility in this field demonstrated low agreement in the evaluation of histologic features [9].

An “orientation tool” among the various histological lesions is, therefore, crucial; it can be obtained by organizing histological lesions in flowcharts according to clinical correlations. Some authors proposed dedicated algorithms for this purpose; two papers proposed a classification system limited to the characteristic of the inflammatory infiltrate [2,10,11], and in another study, the algorithmic approach was limited to the acute colitis patterns [12]. Carpenter et al. proposed a simple diagnostic system based on clinic pathological correlations that included the evaluation of crypt modification and classified colitis histologic patterns in acute pattern and chronic pattern that was subdivided into the crypt destructive pattern and nondestructive pattern [13]. Each pattern corresponds to a limited group of clinical conditions with a specific etiology; in this way, the pathologist is supported in his activity of correlation between morphology and clinic. Crypt destruction is the result of severe, active inflammation of colonic mucosa epithelial structures, which evolves in an irregular regeneration of the same structures (crypt distortion) and a loss of function of bowel mucosa. In our opinion, the occurrence of crypt distortion has a critical role in the diagnostic pathway, as these mucosal lesions identify a group of diseases characterized by chronic, permanent mucosal damage and a disabling clinical course as IBD (destructive colitis) from other inflammatory conditions that are characterized by reversible, transitory damage (nondestructive colitis), with a limited clinical impact.

Furthermore, it must be considered that there are few available studies about the diagnostic performances of pathologists in histologic lesion detection. In a review by Jenkins et al., the histological lesions that favored the diagnosis of IBD were crypt distortion and dense lymphoplasmacytic infiltrate [14]; another author described good reproducibility of a diagnostic system based on a restricted group of histologic lesions [15], but these studies were mainly conducted in a single center. A more recent study from Bentley et al. demonstrated that the number of samples increased the diagnostic performance of pathologists [16].

**Figure 1:**
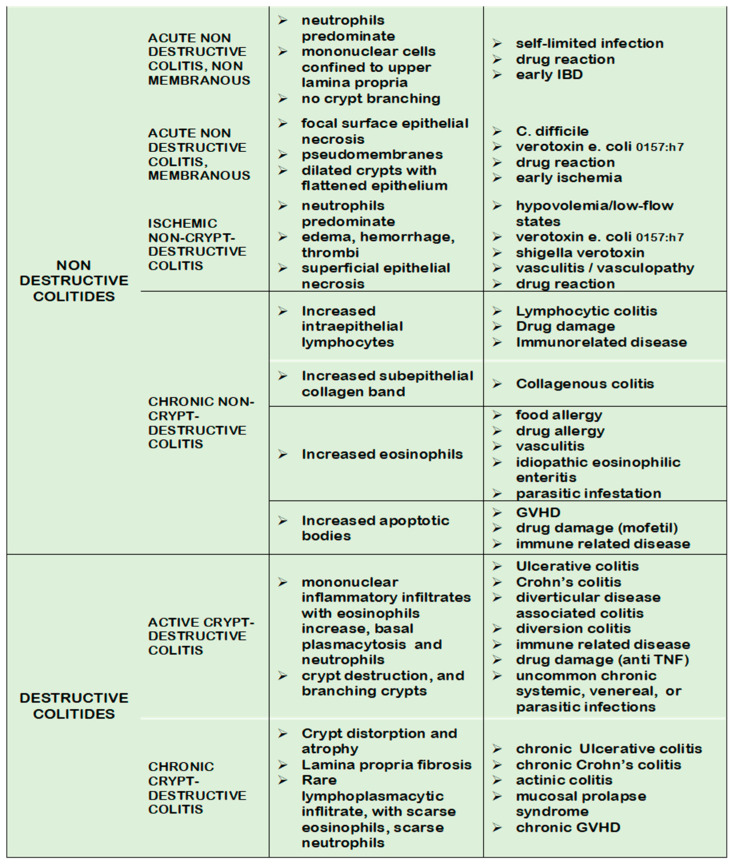
Classification scheme of colitis pattern (From Carpenter et al. [13], modif.).

We planned to explore the applicability and effectiveness of a diagnostic flow chart based on histological patterns and inspired by the scheme of Carpenter [13]. Colitis patterns were categorized into destructive and nondestructive colitis and subsequently subdivided according to the occurrence of intraepithelial neutrophils in bowel mucosa (acute/active inflammation). In the group of nondestructive colitis, a third pattern, based on signs of ischemic damage, was added. In the group of nondestructive inactive colitis, other morphological evidence, such as increased eosinophilic granulocytes, apoptotic bodies, intraepithelial lymphocytes, or basal lamina thickening, is considered for pattern subclassifications (Figure 1).

To acquire data about the topic in clinical practice and to evaluate the interobserver agreement, we conducted a multicenter online survey asking a group of gastroenteropathologists to apply the described classification scheme on digitalized slides from routine colonoscopies performed in our center.

## 2. Materials and Methods

Biopsies from a consecutive series of 20 patients who underwent colonoscopy for suspicion of colitis were retrieved from our archives (years 2017–2021; age range 11–85, mean 51.7, mean n° of sampled sites: 4.1). Eighty-two slides with standard hematoxylin eosin stain from 82 biopsy samplings were digitalized and uploaded into the dedicated open-source E-learning platform. A classification scheme of colitis based on the evidence of crypt modification and, secondly, of active inflammation was conceived, adapted from a format proposed in a previous paper (see introduction) (Figure 1) [8].

**Figure 2:**
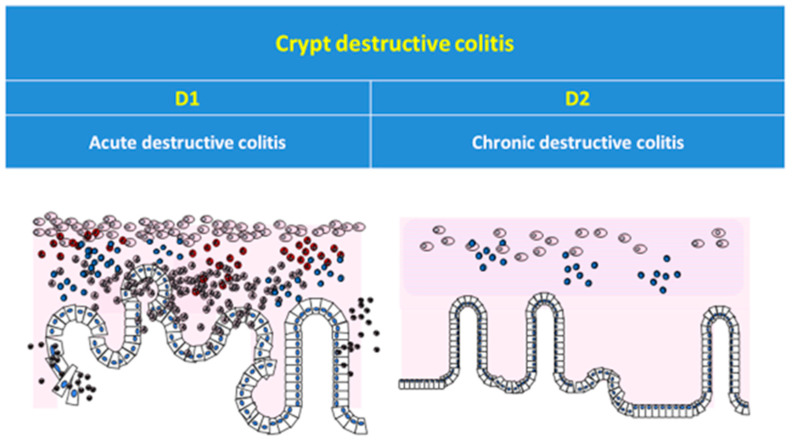

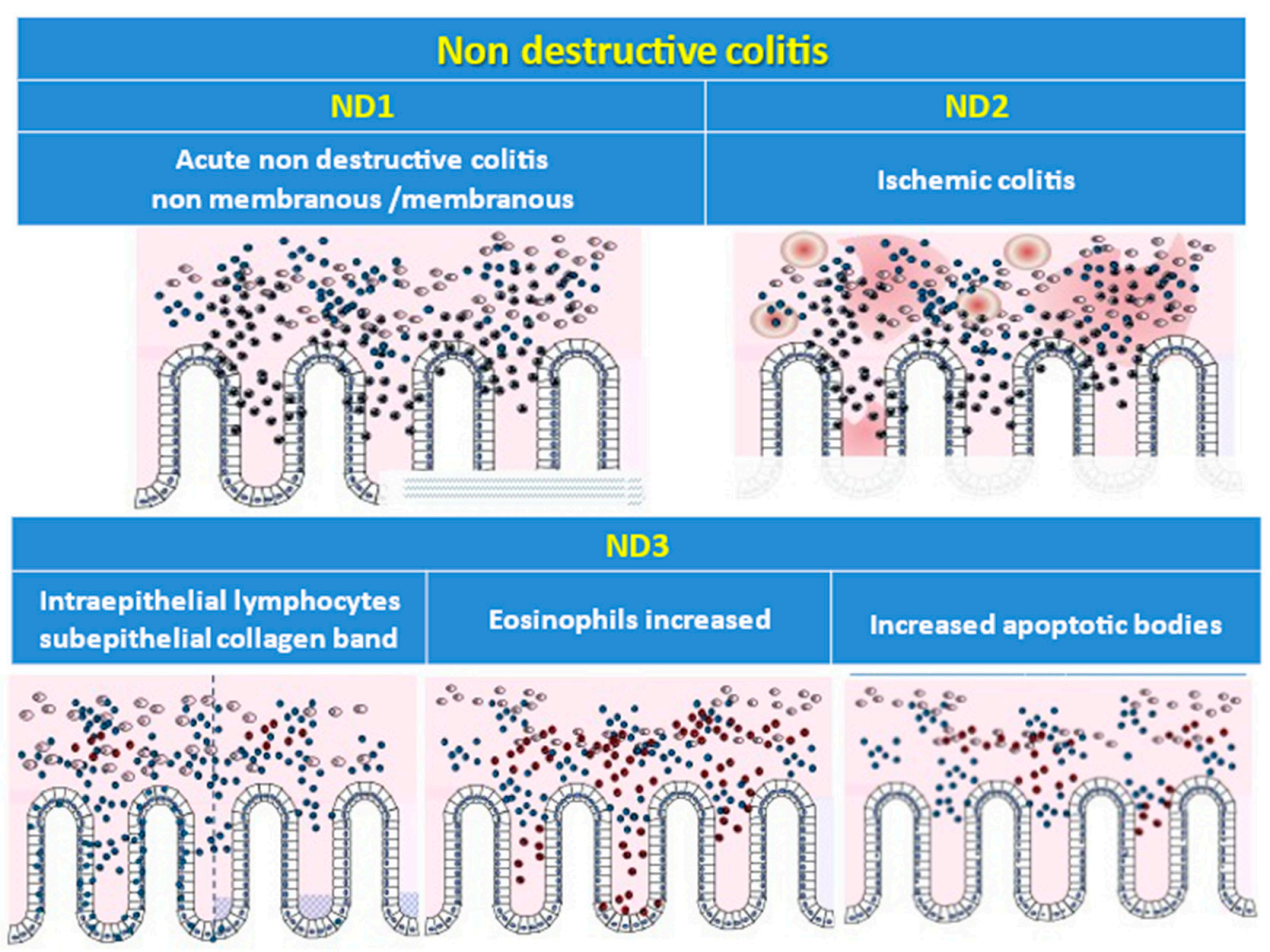
Graphic schemes of pattern classification adopted in the survey.

Twenty pathologists trained in gastroenteric pathology from our region (Piedmont, Italy) were invited for the survey. At first, the morphological patterns included in the scheme adopted for this study were illustrated with a brief clip with graphic renderings. Then, pathologists analyzed the digital slides and answered a series of quizzes with close answers. For each case, the participants were asked to classify the submitted slides according to the main categories of the classification scheme (nondestructive/destructive colitis) and subsequently into their subcategories (ND1-2-3, D1-2) on the basis of the graphic representation of the proposed pattern scheme (Figure 2). The 20 cases were submitted in five sessions from May 2021 to January 2022.

### Statistical Analysis

Data were collected and analyzed with Stata version 12.1 software (StataCorp 2011, College Station, TX, USA). Mean or median values were reported according to data distribution (normal or not normal). To evaluate the agreement among participants, Fleiss’ kappa (k) was calculated. Considering a minimum acceptable kappa (κ0) = 0.6, an expected kappa (κ1) < 0.40, proportion of outcome (*p*) = 0.5, significance level (α) = 0.05 (two-tailed), power (1 − β) = 80%, expected dropout rate = 30%, and the sample size (total of different answers to be analyzed) resulted >= 114. A slight agreement was defined for k values between 0.01 and 0.20, a fair agreement for k values between 0.21 and 0.40, a moderate agreement for k values between 0.41 and 0.60, a substantial agreement for k values between 0.61 and 0.80, and perfect agreement for k values > 0.80.

## 3. Results

Overall, 12 out of 20 participants successfully completed the quizzes related to at least two cases and, therefore, were included in this study. The median number of participants that rated each case was 6 (min 4–max 11); a total of 124 different answers were analyzed (Table 1). In 8 out of 20 (40%) cases, the participants reached full agreement regarding the evaluation of crypt distortion (5 cases: nondestructive colitis; 3 cases: destructive colitis). The calculated agreement was k = 0.432. In the second-level quiz (ND1-2-3 and D1-2), full agreement between participants was achieved for 7 of the 28 (25%) possible classifications, with k = 0.229. Considering responses on both crypt distortion and the corresponding subclassification, no case of complete agreement was observed.

**Figure 3:**
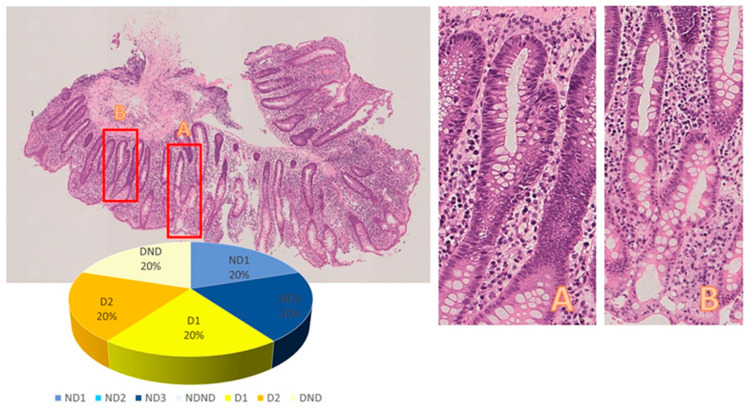
Case 9 Image of a sample from rectum (O.M.: 40×, **left**): Crypt anomalies in frames A and B (O.M.: 200×, **right**) were interpreted as true crypt distortion, and the inflammation was classified as crypt destructive colitis by 60% of the participants. Activity evaluation in this group was controversial (1/3 present, 1/3 absent, 1/3 unclassifiable). Rate of classifications in diagram.

**Figure 4:**
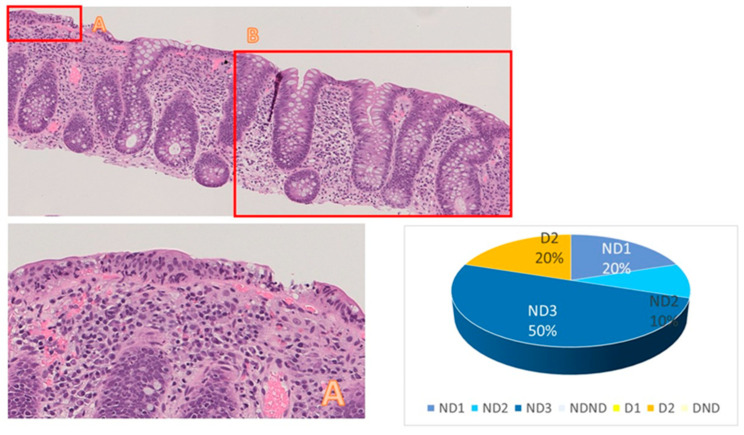
Case 5 Image of a sample from right colon (O.M.: 100×, **upper left**): Crypt anomalies in frame B were interpreted as artefactual, and the inflammation was classified as crypt nondestructive colitis by 80% of the participants and was defined by 50% of the participants as ND3 colitis, with an increase in intraepithelial lymphocytes (Frame A—O.M.: 200×, **lower left**). Rate of classifications in diagram.

Representative slides from two cases analyzed in this study are shown in Figure 3 and Figure 4, where digital frames of the lesions were related to the percentages of responses to the quiz.

## 4. Discussion

Crypt architectural modifications and the occurrence of neutrophils in bowel mucosa (activity) are of paramount importance for the classification of inflammatory conditions with different clinical courses and therapeutic management. Therefore, we believe that accurate and uniform evaluation criteria aimed at identifying these elementary lesions are a fundamental tool for pathologists involved in the diagnosis of non-neoplastic bowel diseases. In the present study, the effectiveness of this diagnostic workup in daily practice was investigated and tested by a digital survey designed to define the interobserver agreement in a pattern classification based on these histological lesions among a group of pathologists trained in gastroenteric pathology. The pathologists were invited to analyze a series of digitalized slides from colonoscopy specimens with a histological diagnosis of colitis and to classify the morphologic characteristics according to a flow chart based on the evidence of crypt modification and active inflammation, adapted from the algorithm proposed by Carpenter et al. [13]. The results of the present study showed a moderate agreement for the evaluation of crypt distortion (k = 0.432) and a fair agreement in pattern subclassification of the two categories (ND1-2-3, D1-2) (k = 0.229).

The findings from this survey, even if limited by the size of the selected cohort, reveal a low consensus about the detection and recognition of the histological lesions that define the pattern classification that was adopted in this study.

The unsatisfactory agreement in the interpretation of the morphological patterns could be explained considering two significant limitations in morphological analysis: (1) the low quality of the histological specimen due to mechanical artifacts, processing artifacts, and inaccurate tissue sectioning and staining procedures; in addition, an adequate amount of sampling should be available for a correct diagnostic workflow. (2) An unsatisfactory definition of the histological lesions in literature: crypt morphologic irregularities due to tissue artifacts must be distinguished from crypt changes due to regeneration consequent to crypt destructive inflammation. Neutrophils in the epithelium should be differentiated from intraepithelial lymphocytes with diapedesis modifications. Evaluation of eosinophils, intraepithelial lymphocytes, and apoptotic bodies that are among the most relevant diagnostic clues of differential diagnosis in the subgroup of chronic nondestructive colitis is often complicated by confounding criteria of evaluation and inhomogeneous cut-off values [17]. In our opinion, pitfalls in pathologists’ interpretations could imply a significant risk of misdiagnosis of bowel inflammatory conditions, hampering the usefulness of histological assessment.

In the field of crypt anomaly evaluations, pathologists’ performances could be improved through an in-depth exploration of crypt arrangement mechanisms during development and in regenerative processes. A series of studies, mainly conducted by Rubio and coworkers, has underlined the importance of crypt asymmetrical branching of regenerating crypts after chronic neutrophil-driven damage as an instrument for a more precise definition of crypt distortion [17,18]. Recently, the same group demonstrated that specimen orientation could represent a bias in crypt morphology evaluation; a significant portion of the histologic section that resulted in a diagnosis of IBD held cross-cut crypts, an inappropriate sectioning plane that modifies the correct assessment of the other histological parameters that defined the diagnosis of IBD [19,20,21]. On the other side, a series of crypt-associated anomalies were described in addition to crypt branching in crypt destructive inflammations that should be considered in their assessment on histologic slides [22]. These new acquisitions could contribute to the construction of more objective criteria for the differential diagnosis between crypt destructive and nondestructive colitis.

Considering neutrophils exocytosis, we could not find in the literature definite criteria to distinguish these elements from intraepithelial lymphocytes or apoptotic bodies, especially when they are limited to isolated elements in the epithelium. Moreover, the minimum number of neutrophils required to establish the occurrence of active inflammation is still considered debated, even in a recent position paper [23].

## 5. Conclusions and Recommendations

In conclusion, the survey proves that there is little agreement, even among committed pathologists, on the interpretation of the histological alterations that are regarded as essential lesions in the diagnostic approach to non-neoplastic bowel diseases, both in previously published pattern classification algorithms and in standard differential diagnosis principles. It must be considered that poor applicability of the criteria for defining elementary morphological lesions weakens the efficacy of the histology in the diagnostic setting of the patient and the evaluation of the parameters of response to therapy in the subsequent follow-up.

Adequate and continuous training for pathologists will be essential for improvement in diagnostic quality. Specific on-site educational courses and online webinars should be organized by experts to shape dedicated pathologists. Optimal technical quality of histological slides is another important prerequisite to suitable diagnostic activity, though tight cooperation with technical staff for adequate inclusion, sectioning, and staining procedures of samples is mandatory for an accurate histological diagnosis. On the other side, scientific societies should continue and strengthen their efforts in providing consensus documents with simple and reproducible criteria for the recognition of the main lesions driving the diagnostic process and uniform cut-off values for cell counts. Immunophenotyping, as well as molecular characterization of inflammatory infiltrate and enterocytes, will probably take part in the diagnostic process in the future, but the advances in basic research still have little impact in routine practice due to the difficulty of applying these techniques on non-neoplastic tissues.

However, we are convinced that histological diagnostics in this field cannot progress outside of a multidisciplinary context; the quality of histological evaluation should be tested through constant comparison with data relating to patient follow-up. To achieve this, it is necessary to institute IBD units in which pathologists trained in the field can collaborate with other specialists in the clinical management of the patient and can follow the clinical evolution of the patients with consequent improvement in diagnostic skills.

## Figures and Tables

**Table 1:** Number of responses per category (ND vs. D) and related subcategory (ND1-2-3, D1-2) for each patient rated by pathologists.

Patient ID	Category	Subcategory				Category	Subcategory		
	ND	ND1	ND2	ND3	Undefined	D	D1	D2	Undefined
1	11	3	1	7	0	0	0	0	0
2	9	0	9	0	0	2	1	1	0
3	0	0	0	0	0	7	2	4	1
4	9	2	0	7	0	0	0	0	0
5	8	2	1	5	0	2	0	1	0
6	1	1	0	0	0	7	3	4	0
7	0	0	0	0	0	6	5	1	0
8	0	0	0	0	0	7	5	1	1
9	2	1	0	1	0	3	1	1	1
10	4	1	0	2	1	3	0	3	0
11	2	0	0	1	1	2	2	0	0
12	1	1	0	0	0	5	5	0	0
13	2	1	0	1	0	3	1	2	0
14	4	3	0	1	0	0	0	0	0
15	3	1	0	2	0	0	0	0	0
16	1	0	0	1	0	3	0	3	0
17	4	1	0	2	1	0	0	0	0
18	2	2	0	0	0	3	1	2	0
19	4	1	1	2	0	0	0	0	0
20	2	1	0	1	0	2	0	2	0

Abbreviations: D, destructive; ND, nondestructive.

## Data Availability

The data underlying this article are available in a repository of our Institution and will be shared on reasonable request to the corresponding author.
